# A Coronavirus Outbreak Linked to a Funeral Among a Romani Community in Central Italy

**DOI:** 10.3389/fmed.2021.617264

**Published:** 2021-06-04

**Authors:** Giancarlo Ripabelli, Michela Lucia Sammarco, Fabio Cannizzaro, Carmen Montanaro, Guido Vincenzo Ponzio, Manuela Tamburro

**Affiliations:** ^1^Department of Medicine and Health Sciences “Vincenzo Tiberio”, University of Molise, Campobasso, Italy; ^2^Department of Prevention, Molise Regional Health Authority, Campobasso, Italy

**Keywords:** COVID-19, epidemiological surveillance, ethnic groups, outbreak, SARS-CoV-2

## Abstract

**Background:** The epidemic dynamics of COVID-19 in the Molise region, central Italy, has dramatically changed from the beginning of May 2020, which was when infections were reported amongst Romani people. The aims of this study were to describe the characteristics of an outbreak that occurred in the Romani community and the interventions implemented for control.

**Methods:** A retrospective analysis of outbreak data was performed to describe the SARS-CoV-2 transmission dynamics.

**Results:** A young Romani woman was the first case reported and epidemiological investigation established a possible link with the funeral of a deceased member of this community. In total, 150 close contacts within 34 family groups in two cities were traced, and 109 (72.7%) Romani individuals were found to be infected by COVID-19. The patient's median age was 31 years, 58% were female, and the highest (20.2%) incidence occurred in the 0–9 years age group. A total of 26 (23.8%) patients developed typical SARS-CoV-2 symptoms, 15 (57.8%) were hospitalized, and 21 (22.1%) had comorbidities [most commonly hypertension (28.6%) and/or coronary heart diseases (23.8%)]. The outbreak was effectively controlled through compulsory quarantine and enhanced active surveillance.

**Conclusions:** This is the first study providing insight into COVID-19 transmission dynamics among a Romani population living in Italy. These findings support general conclusions about the role of crowded social gatherings in SARS-CoV-2 spread, the high communicability among close contacts and household settings, and the impact of asymptomatic carriers. These features are of relevance to certain Romani customs where family gatherings are a fundamental pillar of their lives. Although difficulties emerged in interacting with Romani people related to cultural drivers, beliefs, and lifestyle, the outbreak management was effective and should be considered as a valuable model applicable to similar incidents occurring in minority populations.

## Introduction

As a consequence of a rapid and sudden increase in the number of COVID-19 infections in the Lombardy region (1), the Italian health authorities implemented strict containment measures, including quarantine “red zones” in northern Italy in February 2020 (2). On March 10, 2020, due to the rapid escalation of the epidemic in all Italian regions, lockdown measures were extended to the entire nation (3), which were effective until May 3, 2020 (4).

Molise is a small Italian region of ~300,000 inhabitants, located in central Italy. By May 6, 2020, the regional epidemiological trend of COVID-19 infections with typical symptoms (5) was characterized by a low incidence (99.5 cases/100,000), 7.2% case-fatality rate, 2.9% hospitalization rate, and 22 deaths. The epidemic curve showed a gradual increase of cases except for three major peaks (Table 1), which have been readily responded to the local healthcare infrastructure. On May 7, 2020, 22 new cases were reported by the regional health authority, of which 21 belonged to a Romani community living in Campobasso, the regional capital.

**Table 1:** Results of local COVID-19 surveillance from March to May 2020 in Molise, Italy.

**Date**	**COVID-19 cases**	**Swabs tested**	**Clusters identified in the period**	**Cases (% on the total cases) identified within the Romani community**	**Regional incidence per 100,000 inhabitants**
03/03/20	3	13	None	0 (0)	0.99
04/03/20-30/04/20^*^ (27 days)	295	6,433	Private hospital Nursing home A Nursing home B^#^	0 (0)	96.66
01/05/20-05/05/20^§^ (5 days)	3	1,082	None	2 (66.7)	0.99
06/05/20-27/05/20^§^ (22 days)	133	6,101	Romani community	107 (80.5)	44.00
**Total**	**434**	**13,629**		**109 (25.1)**	**143.58**

* *The lockdown period started on March 9, 2020 in Italy until May 3;*

§ *The reopening phase commenced from May 4, although inter-regional travel was not yet allowed before June 4;*

# *According to Decree of the President of the Council of Ministers on March 8, 2020, the access for relatives and visitors in hospitals and nursing homes or long-term facilities was prohibited since March 9, and only limited to the cases indicated by the Directive of the healthcare structure, strictly required to adopt all the measures needed to prevent any possible transmission and spread of infection; in bold, total number of COVID-19 cases, total swabs tested, total number of cases (%) identified among Romani people, and incidence per 100,000 in Molise region until May 27, 2020*.

The Romani (also spelled Romany), colloquially known as Roma, are an Indo-Aryan ethnic group who mostly live in Europe, originating from northern India, and they have a traditional nomadic itinerant lifestyle.

A local epidemiological investigation was rapidly undertaken, establishing a possible causal link with the funeral of a member of this community, which took place April 30, 2020. Up to this date, COVID-19 cases had not been reported amongst members of the regional Romani community (Table 1), thus supporting a causal link between the funeral and the outbreak. In Italy, attendance at funerals was prohibited from March 8, 2020 (3), but arrangements were relaxed April 26, and then allowed for a maximum of 15 relatives to attend as long as face coverings were worn and social distancing of at least 1m was observed (6).

This study describes the characteristics of an outbreak among the Romani community in the Molise region, the interventions implemented to interrupt the transmission, as well as public health considerations based on their socio-cultural behaviors. To our knowledge, this is the first report of a COVID-19 outbreak in this ethnic group. The effective management and features of this outbreak may be of use as a general model for outbreaks amongst minority communities.

## Methods

This study was a retrospective analysis of the confirmed cases attributable to the outbreak; the data described were anonymously encoded and provided by the department of prevention of the regional health service, which carried out the epidemiological investigation, the contact tracing, the monitoring of molecular testing, and follow up of the cases by a telephone interview.

The COVID-19 case definition was derived from the official documents of the Italian Government (DPCM – Decree of the President of the Council of the Ministers), according to the recommendation of the Italian National Health Institute (ISS), European Centre for Disease Control (ECDC), Centers for Disease Control and Prevention (CDC), and the World Health of Organization (WHO), and comprised the detection of the SARS-CoV-2 by reverse transcription (RT) PCR in a nasopharyngeal swab (7). The outbreak was defined as occurring in the Romani population, or linked with this ethnic group, in the Molise region from May 7 to May 26, 2020; the definition included both primary cases (until 14 days from the initial case detected), as well as secondary cases likely due to the household transmission.

No interventions were performed amongst the individuals included in the present study, above that for the public health controls applied throughout the Molise region by local authorities, and approval by an Ethics Committee was not required. A signed consent form was not obtained because no personal/specific and/or medical information about any identifiable living individuals was reported. For children and adolescents, the local health service acquired information from their parents.

All the individuals identified as cases or close contacts were registered, traced, and followed up by telephone interview by the local health authority according to the guidelines for SARS-CoV-2 contact tracing provided by the Italian Minister of Health. Information was collected on age, gender, general health status regarding previous or concurrent comorbidities, and, where relevant, date of onset of symptoms at diagnosis, severity of symptoms, and outcome. Data were analyzed by IBM Statistical Package for Social Science (SPSS) software version 26.0. Descriptive statistics were used, including means and standard deviation, median, and range for quantitative variables, while counts and percentages were calculated for the qualitative characteristics.

## Results

### Outbreak Description

On the April 30, 2020, ~30 Romani people attended a crowded social gathering devoted to a deceased male belonging to this community who had severe comorbidities: the COVID-19 status of the deceased was unknown, and no pre- or post-mortem swabbing for the virus was performed. A crowded social gathering was likely to have occurred in the house of the deceased in the days preceding death, despite visits were forbidden in the lockdown period. The funeral participation was allowed up to a maximum of 15 relatives observing personal individual protection measures, while a crowded social gathering was reported at the religious ceremony that took place at the cemetery in Campobasso city.

After the funeral, a sudden rise in the incidence of SARS-CoV-2 infections among the local Romani population was detected, and swab testing was soon performed on the members of the local Romani community as well as among people living in the same building as the deceased individual.

**Figure 1:**
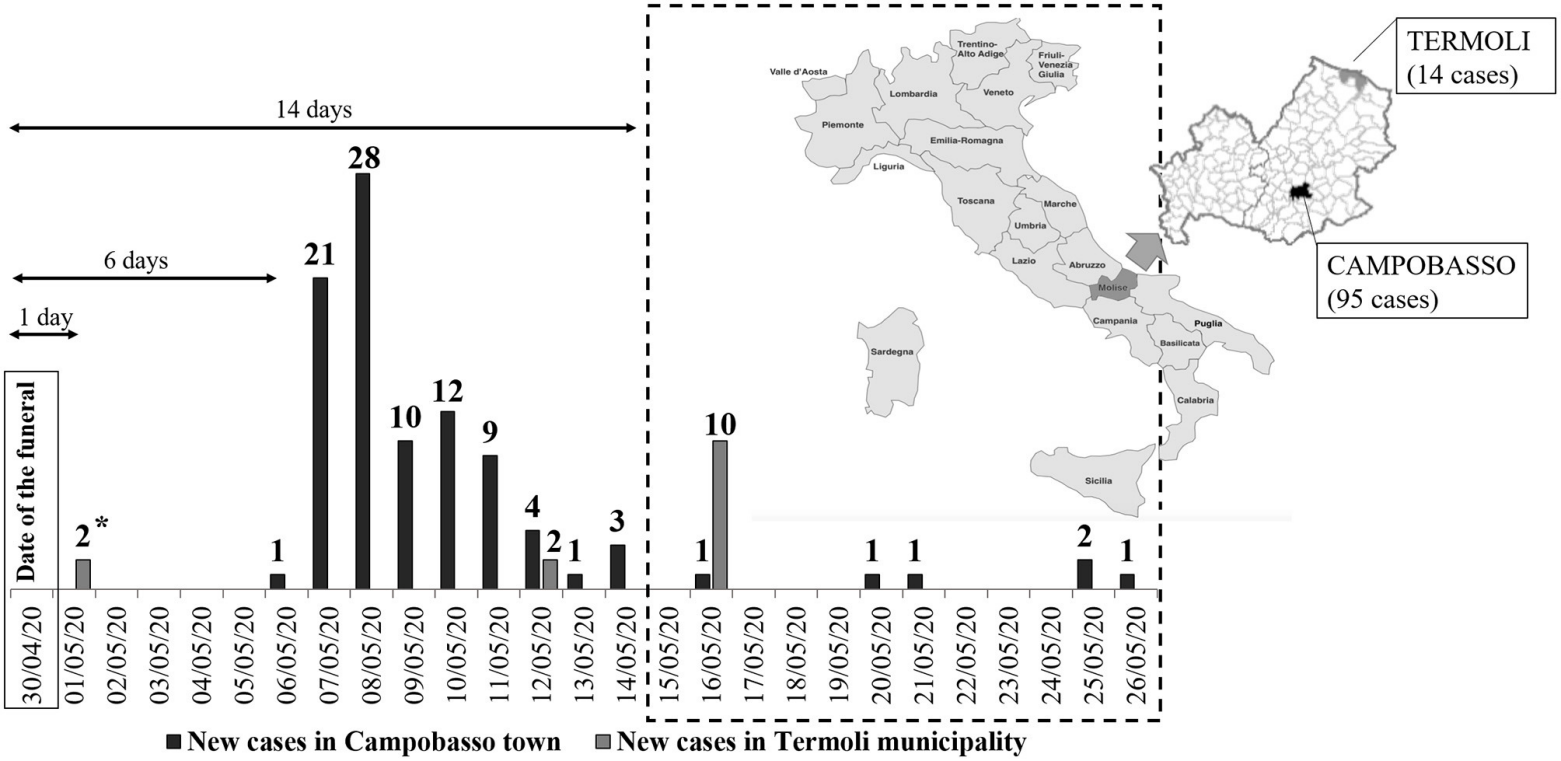
Epidemic curve of COVID-19 cases in the Romani community outbreak in Molise region, central Italy. The arrows indicated time from the presumed exposure, represented by the funeral on April 30, 2020; *cases diagnosed one day after the supposed exposure are likely to be unrelated to the single event of the funeral; the square delimits the 16 secondary cases that occurred between May 15 and 26, 2020, probably due to intrafamilial transmission among the quarantined subjects.

A total of 109 COVID-19 cases occurred in this outbreak, representing 24% of 445 total cases detected in Molise region between March 3 and July 8, 2020. All except 1 of the 109 cases belonged to the Romani community, who were residents in either the main town of Campobasso (95 cases) or in Termoli (14 cases) (Figure 1), a municipality on the coast.

The Romani community living in the Campobasso and Termoli towns consists of ~300 people; therefore, one-third of all the community was involved in the outbreak. In total, 26 (23.8%) individuals developed typical COVID-19 symptoms (5), 15 (57.8%) were hospitalized in the Infectious Diseases ward at the COVID-19 hospital in Campobasso town, and none were admitted to the intensive care. The remaining 83 (76.1%) individuals were asymptomatic. None of the infected patients died.

The first case detected was a Romani person aged between 20–29 years living in Campobasso and who likely attended the burial on the April 30. On the May 5, the patient was admitted to the COVID-19 hospital of Campobasso suffering from symptoms of dyspnea, chest tightness, headache, ageusia, anosmia, and fever. SARS-CoV-2 was detected in a nasopharyngeal swab on May 6, 2020. The epidemic curve included 88 additional COVID-19 cases who were residents in Campobasso town, and were diagnosed within 8 days (up to May 14) from the first case (Figure 1). Cases were also detected in the Romani community in Termoli town with the virus detected in two nasopharyngeal swabs on May 1, which was 1 day after the funeral. In Termoli, 2 and 10 cases were further diagnosed on May 12 and 16 (Figure 1), respectively. There was no information available about familial relationship with the deceased, or whether they had visited the deceased patient in the days preceding death or had attended the funeral. Between May 14 and 26, six cases were further diagnosed in Campobasso.

### Epidemiological Investigation of the Outbreak and Contact Tracing

Following the identification of the first case, contact tracing activities were rapidly implemented by the department of prevention of the regional health authority: 132 and 18 close contacts were identified in Campobasso and Termoli, respectively, among those within the wider family of the first case or those who lived in the same building as the deceased or had likely attended the funeral. Between May 6 and 26, SARS-CoV-2 was detected in 94 (71.2%) contacts in Campobasso and 12 (66.7%) in Termoli. After the first case on May 6, 75% (*n* = 71) of the Romani individuals in Campobasso were identified as being infected with COVID-19 between May 7 and 10, while cases in Termoli mostly occurred between the May 12 and 16. A mandatory quarantine of 14 days was imposed for all traced contacts, including individuals in whom SARS-CoV-2 was not detected.

Analysis of the case contacts allowed identification of 32 Romani familial groups in Campobasso and 2 in Termoli, where one or more family members were identified as being infected with SARS-CoV-2 in 28 (87.5%) family groups in Campobasso town, while three and nine subjects were infected in the 2 groups in Termoli. Within these groups, on average, 82.0% of the family members became infected, with a range varying between 14.3 and 100%. Furthermore, six infected subjects (two family groups) lived in the same building as the deceased in Campobasso, five of which were of the Romani community and one was non-Romani.

### Demographic Characteristics and Underlining Disorders of COVID-19 Patients Within the Outbreak

All except 1 of the 109 cases in the outbreak belonged to the Romani community. The non-Romani adult patient lived in the same building as the deceased individual, but no information on participation in the funeral was available. In total, 57.8% (*n* = 63) of the infected patients were female, and this distribution was observed both for the Campobasso and the Termoli cluster (Table 2). Furthermore, the highest incidence (*n* = 22, 20.2%) of COVID-19 cases occurred in the 0-to-9 years age group (Table 3). Comorbidities or other underlining conditions were self-reported by 19.3% of the COVID 19 cases in Campobasso: the most frequently reported disorder was hypertension in 28.6% (median age: 48 years) of individuals, followed by 23.8% coronary heart diseases (median age: 69 years; Table 2). Symptomatic individuals were male (*n* = 17/26, 65.4%) aged 40–49 years old (median age: 44 years; range: 1–88 years). Of these, 15 (88.2%) aged 50–59 years old were hospitalized. As of July 8, 2020, there were six (5.5%) subjects still infected within the Romani community, and all were asymptomatic.

**Table 2:** Characteristics of COVID-19 cases within the Romani outbreak.

	**Overall outbreak**	**Campobasso cluster**	**Termoli cluster**
	**109 cases**	**95 cases**	**14 cases**
**Ethnic group**			
Romani	108 (99.1%)	94 (98.9%)	14 (100%)
Non-Romani	1 (0.9%)	1 (1.1%)	–
**Gender**			
Female	63 (57.8%)	54 (56.8%)	9 (64.3%)
Male	46 (42.2%)	41 (43.2%)	5 (35.7%)
**Age**			
Mean ± standard deviation	32.1 ± 21.6 years	32.8 ± 21.5 years	30.3 ± 23.1 years
Median	31 years	33 years	25.5 years
Range	1–88 years	1–88 years	1–83 years
**Comorbidities or underlining conditions**			
Hypertension	6 (5.5%)	6 (6.3%)	–
Coronary heart diseases	5 (4.6%)	5 (5.3%)	–
Breast cancer	1 (0.9%)	1 (1.1%)	–
Frail elderly with multiple pathologies	1 (0.9%)	1 (1.1%)	–
Hepatitis B	1 (0.9%)	1 (1.1%)	–
Microcytic anemia	1 (0.9%)	1 (1.1%)	–
Hiatal hernia	1 (0.9%)	1 (1.1%)	–
Aphonia/dysphonia	1 (0.9%)	1 (1.1%)	–
Psoriasis	1 (0.9%)	1 (1.1%)	–
Celiac disease	1 (0.9%)	1 (1.1%)	–
Neurofibromatosis	1 (0.9%)	1 (1.1%)	–
Chronic colitis	1 (0.9%)	1 (1.1%)	–

**Table 3:** Age-based frequency of COVID-19 cases before, during, and after the Romani outbreak compared to the whole regional and national data.

**Age group**	**Cases (*n* = 296) in Molise before Romani outbreak (Period 3^**rd**^ March−28^**th**^ April) (28)**	**Cases (*n* = 109) within the Romani outbreak (Period 6^**th**^-26^**th**^ May)**	**Cases (*n* = 422) in Molise after Romani outbreak (Period 3^**rd**^ March−20^**th**^ May) (25)**	**Cases (*n* = 199,470) in Italy (Period 20^**th**^ February−28^**th**^ April) (28)**
0–9 years	1.4%	20.2%	5.5% ↑	0.7%
10–19	4.7%	9.2%	6.2% ↑	1.3%
20–29	4.4%	15.6%	8.8% ↑	5.2%
30–39	10.5%	19.2%	12.6% ↑	7.5%
40–49	11.8%	13.8%	12.3% ↑	12.9%
50–59	25.0%	9.2%	21.6% ↓	18.0%
60–69	17.9%	7.3%	14.0% ↓	14.0%
70–79	8.8%	2.7%	6.9% ↓	15.1%
80–89	10.1%	2.7%	8.3% ↓	17.7%
≥90	5.4%	0.0%	4.0% ↓	7.6%

*The arrows (↑ and ↓) indicated the increase or decrease of the frequency in the stratified age groups as consequences of the Romani outbreak*.

### Intervention Measures

The regional health authority required the reinforcement of local surveillance around the Romani community's residences in agreement with the police, to assure full compliance for control. Furthermore, on May 9, 2020, both the Mayors of Campobasso and Termoli issued ordinances to enhance the control activities to specific areas to allow the enforcement of isolation at home. During the period between May 6 and May 26 (dates from the first case being detected to the last case diagnosed within the regional Romani community), swab testing significantly increased to a total of 13,452 with a daily average of 282.1 ± 156.3. Soon after the rapid increase in cases, quarantine was established for 14 days after close contact with cases and was maintained until a virological negative result was obtained. Additionally, for outbreak management, timely effective risk communication was a specific task of the department of prevention of the regional health authority with a “culturally adapted” and context-specific strategy focusing on restrictions to minimize crowded social gatherings (which occur frequently in this particular ethnic group), as improving stay-at-home policies and hygiene measures in the household setting. The risk communication was carried out based on explanation and reiteration by telephone to both the householders and heads of the Romani community about the rules to be respected. Furthermore, the local authorities talked to them constantly about these issues and guaranteed all the needs to support them (i.e., foods and medicine). Between March 4 (the day after the first three SARS-CoV-2 cases detected in our region) and April 30, 2020 (date of the funeral of Romani subject), no SARS-CoV-2 cases were identified among the Romani population out of a total of 6,433 swab tests performed at the regional level (Table 1) including those performed on residents/workers in nursing homes or hospitals and in the general population. During this period, SARS-CoV-2 clusters occurred in nursing homes, hospitals, or long-term facilities, where, as of March 9, according to the Decree of the President of the Council of the Ministers, the access for relatives and visitors was prohibited and limited only to the cases indicated by the Directive of the healthcare structure, who was strictly required to adopt all the measures needed to prevent any possible transmission and spread of infection.

## Discussion

The Romani community has its own lifestyle and rules that may not coincide with those of non-Romani citizens (9). Their society has a horizontal organization, with a central role of the family; contacts are extremely frequent between members, and great support is offered at the end of the life (10) since death is considered an important event reinforcing family and community bonds (11). Empathy for someone who is dying is one of the strongest drivers of their culture, as described for a female Romani leader in Catalonia who died accompanied by more than 200 people (11).

The outbreak described in this study received nationwide attention and generated great concerns. Further cases linked to this outbreak occurred in the Romani communities in some neighboring regions, as individuals came to Molise to pay tribute to the prominent person who passed away. Before April 30, no cases within the Romani community were reported by the Molise health service. The outbreak caused prejudice toward Romani people although they have a good level of integration in the local society, mainly because the stringent national rules on lockdown were still in force but were disregarded at the time of the funeral.

Development of a clear case definition is critical conducting an effective investigation of an outbreak that should be defined as more cases of the disease in time or place than expected, and when two or more cases have the same laboratory diagnosis of the etiologic agent. Hence, criteria for person, place, time, and clinical features should be included in a case definition and should be specific. In the outbreak described here, the cases were identified as resulting from the contact tracing activities carried out by the department of prevention of the regional health service, representing an effective public health measure for the control of COVID-19, enabling prompt identification and management of the contacts of cases, and identifying secondary cases after transmission from the primary cases. Hence, considering contact tracing steps, persons who may have been exposed to SARS-CoV-2 as a result of being in contact with an infected person were identified; these contacts were traced, and information about suitable infection control measures, symptom monitoring, and other precautionary measures was provided.

Contact tracing remains of fundamental importance to delimitate clusters of infection, although the interactions with Romani people were difficult due to their social and behavioral rules.

The epidemiological investigation allowed identifying the outbreak, which was likely to have been linked to the participation and crowded social gathering of people together at the funeral or attending the body of the deceased before leaving home to be buried. However, the outbreak could not only be associated with participation in these ceremonies as well as to the presence of an initial infection “super spreader,” because it should be also considered the likelihood that many individuals visited the moribund in the days preceding death, with the funeral possibly amplifying a pre-existing viral circulation within the community.

Indeed, if the exposure was the funeral on April 30, this should exclude the two cases in Termoli town who tested positive on May 1 (Figure 1), which were counted as related to the outbreak by the local health authority since they were within the Romani community. These subjects denied having attended the funeral, and no information was available about familial relationship with the deceased, or whether they had gone to the home of the deceased in the days preceding death. We hypothesize that these two cases could reflect the virus circulating within the Romani community prior to the funeral but could also represent “patient zero” as well as an alternative source of disease transmission for the cases diagnosed on May 12 in the Termoli cluster (Figure 1). As reported by the regional health authority, the first case was diagnosed on May 6, a total of 5 days after the two cases reported in Termoli and 6 days after the funeral, in line with the estimated median incubation period of 5–6 days (12–14). Following the major peak on May 8 (Figure 1), 16 cases were diagnosed after May 14, which are likely to represent intrafamilial transmission amongst quarantined individuals in Campobasso: the secondary transmission rate was estimated as of 16.8%.

For an in-depth investigation of the outbreak, it is important to report the geographic distribution of the identified cases. Based on the available information, the regional Romani community resides in Campobasso (the capital city), as well as in the towns of Isernia and Termoli. Anyway, the outbreak described was composed only of two clusters of infections, diagnosed in Campobasso and Termoli.

This outbreak demonstrated the high communicability of COVID-19, which was evidenced by the secondary cases identified among household contacts and is in agreement with the available reports for COVID-19 (12, 15, 16). It has been estimated that the basic reproduction number (R0) for COVID-19 ranged between 2 and 3.5 (17). Certainly, the transmission rate can be much higher in closed and crowded social gatherings (18), such as funerals that have been linked to the spread of other diseases (19). Furthermore, COVID-19 community transmission decreased over time, while household transmission increased under stay-at-home policies, leading to a secondary attack rate of 16.3% in the household context (20).

The outbreak described here confirms that household transmission is of high concern, underlining the need to implement appropriate management strategies. To control the outbreak, local authorities suggested transferring Romani people to locations different from their houses but disregarded this option. In the COVID-19 transmission, isolation at home is significant, especially for this population where overcrowding is common, and this is a well-recognized risk factor associated with various health problems, including respiratory infections (21).

In comparison to available Italian national data where 54.2% of COVID-19 cases were identified amongst females (22), this outbreak showed a higher incidence of 57.8% among females, which may reflect differences in social and behavioral habits amongst the Romani community (23). Globally, the ongoing COVID-19 pandemic is affecting the whole population, although different susceptibilities have been reported (24), considering males, old age, and those with comorbidities (mainly hypertension, diabetes, and cardiovascular disease) as the most important risk factors for severe symptoms and outcome (13, 25).

An additional key epidemiological feature of the Romani outbreak concerned the younger age of infected individuals (median age 31 years) compared to the total regional (median age 52 years) and national cases (median age 62 years) (26). This finding may be explained by the lower age of Romani people compared to the general population (21); the poor perception of younger people of COVID-19 risks as compared to older people (27), and because swab testing was extended to detect cases in this particular ethnic group, which is generally younger than the regional population, consisting of 25% of individuals aged ≥65 years. Estimates of the relative COVID-19 illness ratio revealed age-related increases in Spain and Italy, while higher ratios among middle or younger age groups were reported in China and Korea (28), probably due to more extensive swab testing.

The age-related frequency of COVID-19 cases in the Molise region underwent marked changes due to the outbreak described here, especially for the 0-to-9-year age group, accounting for 1.4% and 5.5% of cases before and after the outbreak (8, 26), respectively, being 20.2% in the infected Romani population. Hence, children were the most frequently infected group, in contrast to national data where only 0.9% cases occurred in the 0-to-9-year age group (Table 3).

In Italy, 34.3% of COVID-19 cases occurred in subjects with at least one co-existing/underlining pathologies, including cardiovascular, respiratory, metabolic, cancer, or other chronic diseases (29). Based on the data available, 19% of Romani cases living in Campobasso town declared pre-existing pathologies, and heart diseases were common together with other chronic diseases, according to previous reports on this population group (30). However, the health status of this group remains largely unknown as does the distribution of disease-associated predisposing factors (31). The cases that occurred in Termoli did not report any underlining condition, and this finding is in agreement with previous evidence indicating that Romani people self-report better individual health status than the rest of the population due to the lower age, different health perceptions, and difficulties in accessing and receiving proper healthcare (32).

In conclusion, about a quarter of COVID-19 cases registered until May 2020 in the Molise region of Italy were related to the outbreak described here, which highlights the major role of crowded social gatherings in the spread of COVID-19 and the importance of transmission through asymptomatic individuals, including children. This is in line with data obtained through the national seroprevalence survey, which indicated that SARS-CoV-2 IgG positive individuals six times more than the total number of officially diagnosed cases through viral RNA detection (33).

The Romani population represents the largest ethnic minority group in Europe for which an increased vulnerability to COVID-19 pandemic faces a combination of health risks, leading the Council of Europe to issue a statement on the need for governments to ensure equal protection and care for this community (34). Difficulties related to socio-cultural barriers were encountered when trying to make contact with and acquire information from the Romani people involved in this outbreak. In such situations, facilitators and interventions to increase active participation in public health strategies should be identified and tailored for this population, as well as for “hard to reach” minority groups (35). Taking into account these difficulties, the response to the outbreak was effective in limiting further transmission and containing the cases. Hence, outbreak management can be considered as a general model for similar events amongst specific ethnic groups, particularly where there are health inequalities.

This study has some limitations, including the total number of Romani attending the funeral that could not be accurately ascertained, which might have over-estimated transmission level linked to this specific event. In addition, although the surveillance and contact tracing were rigorously implemented, some cases that occurred outside the Romani community could have been missed. Nonetheless, the study has noteworthy strengths. To our knowledge, no other study reported COVID-19 infections amongst ethnic Romani groups either nationally or internationally. Hence, this is the first report that provides insights into COVID-19 epidemiology, transmission dynamics, and control measures of crowded intrafamilial gatherings while continuing to deal with the pandemic. This study has great relevance to epidemiological findings and surveillance features: first, the younger age of the infected people involved in the outbreak as compared to the regional and national cases reported, and considering lifestyles, traditions, and viewpoint on health status, which significantly differ in Romani communities compared to the general population. Moreover, the peculiarity of this study relies on a well-defined ethnic population, with their own behavior and familial practices, providing an example for an adequate response, which can differ from normal management procedures due to their cultural and social characteristics.

The outbreak had important implications also at the regional level even in the non-Romani population, because it occurred in the reopening phase following the national lockdown, generating serious concerns among the general population. To stop the propagation of the outbreak, it was necessary to reinforce the surveillance, which had an impact on the whole regional territory. Similarly, the enhanced communication about prevention measures to contain the transmission of the outbreak among Romani people, mainly avoiding crowded social gatherings, especially between families, had a huge influence on the general population as well.

This outbreak led to particular public health considerations, which could have been further investigated by phylogenetic analysis of the viral strains involved in this outbreak for comparison with those circulating locally within Molise region, as well as nationally and internationally.

## Data Availability Statement

The datasets presented in this article are not readily available because, all data are already described in the study. Requests to access the datasets should be directed to ripab@unimol.it.

## Ethics Statement

Ethical review and approval for the study on human participants was not required in accordance with the local legislation and institutional requirements. A signed consent form was not obtained because no personal/specific and/or medical information about any identifiable living individuals was reported, and the study was based on a retrospective analysis of outbreak data. For children and adolescents, the local health service acquired information from their parents.

## Author Contributions

GR designed the study and contributed to data interpretation and critically editing the report. MS and FC performed the literature search, data organization, and result interpretation. CM and GVP contributed to data collection and encoding. MT led the principal analysis of the available data and wrote the draft and edited the manuscript. All authors read and approved the final report.

## Conflict of Interest

The authors declare that the research was conducted in the absence of any commercial or financial relationships that could be construed as a potential conflict of interest.
